# Down-regulation of HCP5 inhibits cell proliferation, migration, and invasion through regulating EPHA7 by competitively binding miR-101 in osteosarcoma

**DOI:** 10.1590/1414-431X20209161

**Published:** 2021-01-08

**Authors:** Yangmao Tu, Qing Cai, Xuemei Zhu, Min Xu

**Affiliations:** 1 Department of Orthopedics, Jingzhou Hospital of Traditional Chinese Medicine, Jingzhou, China; 2 Jingzhou Hospital of Traditional Chinese Medicine - Functional Section, Jingzhou, China; 3 Jingzhou Institute of Technology, College of Textile and Apparel Design and Art, Jingzhou, China

**Keywords:** lncRNA HCP5, Osteosarcoma, miR-101, EPHA7

## Abstract

Patients with osteosarcoma (OS) usually have poor overall survival because of frequent metastasis. Long non-coding RNAs (lncRNAs) have been reported to be associated with tumorigenesis and metastasis. In this study, we investigated the expression and roles of lncRNA human histocompatibility leukocyte antigen (HLA) complex P5 (HCP5) in OS, aiming to provide a novel molecular mechanism for OS. HCP5 was up-regulated both in OS tissues and cell lines and high expression of HCP5 was associated to low survival in OS patients. Down-regulation of HCP5 inhibited cell proliferation, migration, and invasion, suggesting its carcinogenic role in OS. miR-101 was targeted by HCP5 and its expression was decreased in OS. The inhibitor of miR-101 reversed the impact of HCP5 down-regulation on cell proliferation, apoptosis, and metastasis in OS. Ephrin receptor 7 (EPHA7) was proved to be a target of miR-101 and had ability to recover the effects of miR-101 inhibitor in OS. In conclusion, lncRNA HCP5 knockdown suppressed cell proliferation, migration, and invasion, and induced apoptosis through depleting the expression of EPHA7 by binding to miR-101, providing a potential therapeutic strategy of HCP5 in OS.

## Introduction

Osteosarcoma (OS) is an osteoid-producing high-grade malignancy of bone of mesenchymal origin (1). Due to the high hereditary instability of OS cells, extensive heterogeneity of histology, lack of biomarkers, localized intense aggressiveness, and rapid metastatic potential (2), OS therapy has produced little prognosis improvement over the past 20 years. Survival rates of patients with metastatic disease or relapse continue to be unsatisfactory (1). Therefore, understanding of the OS biology remains a complex challenge. The purpose of this paper was to provide a new perspective on the tumorigenesis and metastasis of OS.

Increasing evidence has suggested that non-coding RNAs have important roles in occurrence and progression of cancer (3,4). Long non-coding RNAs (lncRNAs), longer than 200 nucleotides, are defined as RNA molecules that do not translate proteins (5). Human histocompatibility leukocyte antigen (HLA) complex P5 (HCP5) belongs to lncRNAs, and numerous reports have studied the functional roles of lncRNA HCP5 in cancers. For example, HCP5 acted as a competing endogenous RNA (ceRNA) to activate alpha-2,6-sialyltransferase 2 (ST6GAL2) by targeting microRNA (miR)-22-3p, miR-186-5p, and miR-216a-5p, and promoted the proliferation, migration, invasiveness, and angiogenic ability of follicular thyroid carcinoma cells (6). HCP5 up-regulates runt-related transcription factor 1 (RUNX1) by acting as miR-139 sponge to promote astrocyte elevated gene-1 (AEG-1) expression, which is involved in several oncogenic effects in glioma cells (7). Overexpressed HCP5 promotes the tumorigenesis of cervical cancer by enhancing metastasis associated in colon cancer-1 (MACC1) expression by acting as miR-15a sponge (8). These data reveal that HCP5 plays a circle role in the development of different cancers. However, little study of HCP5 in OS has been reported.

MicroRNAs (miRNAs) are a class of non-coding RNA molecules and regulate gene expression by binding to target mRNAs, leading to mRNA translational inhibition or degradation (9). miRNAs continually participate in diverse diseases because of the occurrence of genomic events, such as mutations, deletion, amplification, transcriptional changes, biogenesis mutations, or the down-regulation of enzymes that regulate miRNAs biogenesis (10–13). Recently, the study of miRNAs on cancer has made tremendous progress. It has been identified that miR-101 participates in certain types of cancers. Huang et al. (14) showed that miR-101 was reduced in diffuse large B cell lymphoma and correlated with tumor formation and progression. Chu et al. (15) reported that it was highly up-regulated in liver cancer and inhibited the activation of NF-κB and MAPK pathways through sponging miR-101. Gonzales et al. (16) suggested that miR-101 acted as a tumor suppressor in mixed-lineage leukemia-rearranged acute myeloid leukemia. Therefore, the role of miR-101 in OS is definitely worth further investigation.

Erythropoietin-producing hepatoma-amplified sequence (Eph) receptors are a large family of receptor tyrosine kinases in humans (17). Ephrin receptor 7 (EPHA7), a member of the Eph family, belongs to a subgroup of receptor tyrosine kinases and plays diverse roles in carcinogenesis (18). For instance, EPHA7 was up-regulated in human laryngeal squamous cell carcinoma tissues, and EPHA7 suppression inhibited cell growth and promoted cell apoptosis (19). EPHA7 expression is relatively higher in the non-small cell lung cancer (NSCLC) cell lines, and down-regulation of EPHA7 suppresses the proliferation of NSCLC cells (18). EPHA7 down-regulation induces apoptosis via the intrinsic apoptotic pathway (20). These data suggest the role of EPHA7 in multiple cancers and provide valuable reference for our research, despite the knowledge of the effects of EPHA7 in OS being limited.

In this study, we investigated the expression of HCP5 in OS tissues and cells, explored the role of HCP5 in the regulation of proliferation, migration, invasion, and apoptosis of OS cells, and determined the interaction between miR-101 and HCP5 or EPHA7. The purpose of this paper was to determine a potential mechanism of HCP5 function in OS and provide a theoretical basis for the treatment of OS.

## Material and Methods

### Patients and tissues

A total of 40 patients treated in the Department of Orthopedics, Jingzhou Hospital of Traditional Chinese Medicine were selected for this research, and each of them signed an informed consent before surgery. All OS tissues and adjacent tissues were stored according to standard sample storage method after surgery. This research was approved by the Ethics Committee of the Department of Orthopaedics, Jingzhou Hospital of Traditional Chinese Medicine.

### Cell culture

Human OS cell lines, including MG-63, U2OS, 143B, and HOS, and human osteoblast cell line hFOB 1.19 were all purchased from American Type Culture Collection (ATCC, USA). Dulbecco's modified eagle medium (DMEM) supplemented with 10% fetal bovine serum (FBS; Life Technologies, USA) was used to culture these cell lines in a humidified condition with 5% CO_2_ at 37°C.

### Cell transfection

For functional analysis, small interference RNA (siRNA) against HCP5 (si-HCP5), siRNA negative control (si-NC), pcDNA-HCP5 overexpression vector (HCP5), pcDNA empty vector (vector), miR-101 mimics, miR-101 mimics negative control (miR-NC), miR-101 inhibitor, miR-101 inhibitor negative control (NC inhibitor), and siRNA against EPHA7 (si-EPHA7) were all obtained from RiboBio company (Guangzhou, China). Based on manufacturer instructions, cell transfection was carried out using Lipofectamine 3000 (Invitrogen, USA).

### Quantitative real-time polymerase chain reaction (qRT-PCR)

Trizol reagent (Takara, China) was used to extract total RNA from tissues and cells. RNA concentration was detected by NanoDrop 2000 spectrophotometers (ThermoFisher Scientific, USA). Then, cDNA was synthesized from RNA using QuantiTect Reverse Transcription Kit (QIAGEN, USA). SYBR Green Taq Mix (Takara) was used to measure the expression level under ABI 7500 Thermocycler (ThermoFisher Scientific). The reaction conditions were as follows: 95°C for 5 min, followed by 40 cycles of denaturation at 95°C for 15 s, annealing step at 60°C for 30 s, and extension at 72°C for 90 s. Relative expression was quantified using the 2^-ΔΔCt^ method. GAPDH and U6 were used as internal controls. The primers were as follows: HCP5, F: 5′-ACCACTATTGGCCATCAAAGG-3′ and R: 5′-ATACTGTCCAATTCCCCTGT-3′; EPHA7, F: 5′-CTAATGTTGGATTGTTGGCAAAAG-3′ and R: 5′-TTGATCCAGAAGAGGGCTTATTG-3′; GAPDH, F: 5′-CTGGGCTACACTGAGCACC-3′ and R: 5′-AAGTGGTCGTTGAGGGCAATG-3′; miR-101, F: 5′-CGGCGGTACAGTACTGTGATAA-3′ and R: 5′-CTGGTGTCGTGGAGTCGGCAATTC-3′; U6, F: 5′-CTCGCTTCGGCAGCAGCACATATA-3′ and R: 5′-AAATATGGAACGCTTCACGA-3′.

### Western blot assay

Transfected cells were lysed using radioimmunoprecipitation assay (RIPA) (Beyotime, China) to acquire total protein; the total protein was then separated by sodium dodecyl sulfate-polyacrylamide gel electrophoresis (SDS-PAGE) and electro-transferred onto a polyvinylidenedifluoride (PVDF) membrane (Millipore, China) on ice. Next, the membrane was placed in a blocking solution (phosphate-buffered saline (PBS)) containing 5% nonfat milk for 2 h. The membrane was incubated with primary antibodies (1:1000; Abcam, USA) against EPHA7 at 4°C overnight. The following day, after being washed 3 times with PBS, the membrane was incubated in horseradish peroxidase-labeled secondary antibody at room temperature for 2 h. Finally, protein blot signal was visualized using the enhanced chemiluminescence (ECL) detection kit (ThermoFisher Scientific), and the gray intensity of blots was quantified using ImageJ software (NIH; USA). GAPDH was considered as the internal control.

### Cell proliferation assay

Transfected U2OS and MG-63 cells (5×10^3^) were seeded in 96-well plates for 24 h. After incubation, 0.5 mg/mL 3-(4,5-dimethyl-2-thiazolyl)-2,5-diphenyl-2-H-tetrazolium bromide (MTT) solution (Sigma-Aldrich, USA) was added into each plate and incubated for another two hours. Finally, absorbance at 490 nm was measured at 24, 48, 72, and 96 h by a microplate reader (Bio-Rad, USA). Five replicate wells were used for each group.

### Cell migration and invasion analysis

For cell migration and invasion, transfected U2OS and MG-63 cells were harvested after culture for 48 h. Then, cells were re-suspended in serum-free DMEM and placed into the upper 24-well transwell chamber (Corning, USA) for migration assay or into the upper chamber of 24-well transwell membrane filter coated with Matrigel (Corning, USA) for invasion assay. Meanwhile, DMEM solution mixed with 10% FBS was added onto the lower chamber. After 24 h, the migrated or invaded cells on the lower surface were fixed with 4% paraformaldehyde (PFA) and stained with methanol containing 0.1% crystal violet for 15 min. Finally, five randomly selected fields were used to calculate cell number using an Olympus microscope (Olympus, Japan) at a magnification of ×100.

### Cell apoptosis analysis

Fluorescein isothiocyanate (FITC) Annexin V apoptosis detection kit (Invitrogen) was used to detect cell apoptosis according to manufacturer instructions. In brief, MG-63 and U2OS cells were incubated for 48 h after transfection. Afterwards, cells were washed twice using ice-cold PBS and re-suspended in binding buffer at a concentration of 1×10^6^ cells/mL. Next, 5 μL Annexin V-FITC and PI were added to the cell suspension and stained for 15 min at room temperature in a dark room, followed by addition of 400 μL binding buffer. Subsequently, the apoptotic cells were distinguished using a flow cytometer (BD Biosciences, USA).

### Bioinformatics prediction and luciferase reporter assay

The putative binding sites of HCP5 or EPHA7 and miR-101 were predicted by miRcode (http://www.mircode.org) and TargetScan (http://www.targetscan.org/vert_72/), respectively. Cells were seeded in the 24-well plates for 24 h, then HCP5 wild-type (HCP5-WT) or HCP5 mutant (HCP5-MUT) sequences containing miR-101 binding sites were constructed onto the downstream of pmirGLO vector (Promega, USA). Afterwards, HCP5-WT or HCP5-MUT and miR-101 mimics or miR-NC were co-transfected into OS cell lines. Dual-luciferase assay system (Shanghai Qcbio Science & Technologies Co., Ltd, China) was used to detect luciferase activities of MG63 and U2OS after transfection for 48 h. The 3′ UTR sequences of EPHA7 containing wild-type (WT) or mutant (MUT) binding sites of miR-101 were amplified and cloned into pmirGLO vector (Promega) to generate EPHA7-WT or EPHA7-MUT luciferase reporter vector, respectively. Transfection and detection were performed as described above.

### Statistical analysis

GraphPad Prism v5.01 software (GraphPad Software, USA) was used for statistical analysis. All data are reported as means±SD from at least three independent experiments. Statistical analysis of data was performed using the Student's *t*-test between 2 groups and analysis of variance (ANOVA) among multiple groups. Differences were considered to be statistically significant when P<0.05.

## Results

### HCP5 was up-regulated in OS tissues and cell lines

**Figure 1:**
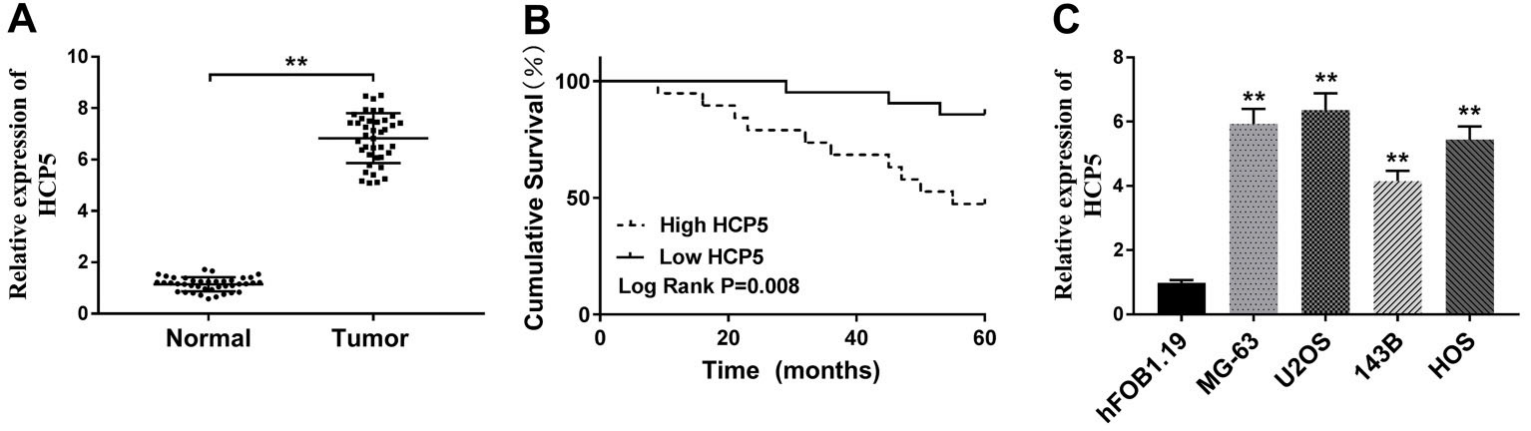
HCP5 was highly expressed in osteosarcoma (OS) tissues and cell lines. **A**, QRT-PCR assay was performed to measure the expression of HCP5 in 40 pairs of OS tissues and adjacent normal tissues. **B**, The cumulative survival of OS patients with high HCP5 (n=19) expression or low HCP5 (n=21) expression was recorded. **C**, Relative expression of HCP5 in different OS cell lines and human osteoblast cell hFOB 1.19 was detected by qRT-PCR. Data are reported as means±SD. **P<0.01 (Student's *t*-test and ANOVA).

First, we evaluated the expression of HCP5 in OS by qRT-PCR. HCP5 expression was significantly increased in tumor tissues compared with adjacent normal tissues (Figure 1A). The Kaplan-Meier method was used to determine the relationship between HCP5 expression and the prognosis of patients. Predictably, the overall survival of patients with low expression (n=21, 85.71%) of HCP5 was significantly higher than in those with high expression of HCP5 (n=19, 52.63%) (Figure 1B). In addition, as exhibited in Figure 1C, there was a significant increase of HCP5 expression in OS cell lines (MG-63, U2OS, 143B, and HOS) compared with normal cells (hFOB1.19). The data suggested that HCP5 played a carcinogenic role in OS and led to poor survival.

### HCP5 down-regulation suppressed cell proliferation, migration, and invasion but promoted cell apoptosis

**Figure 2:**
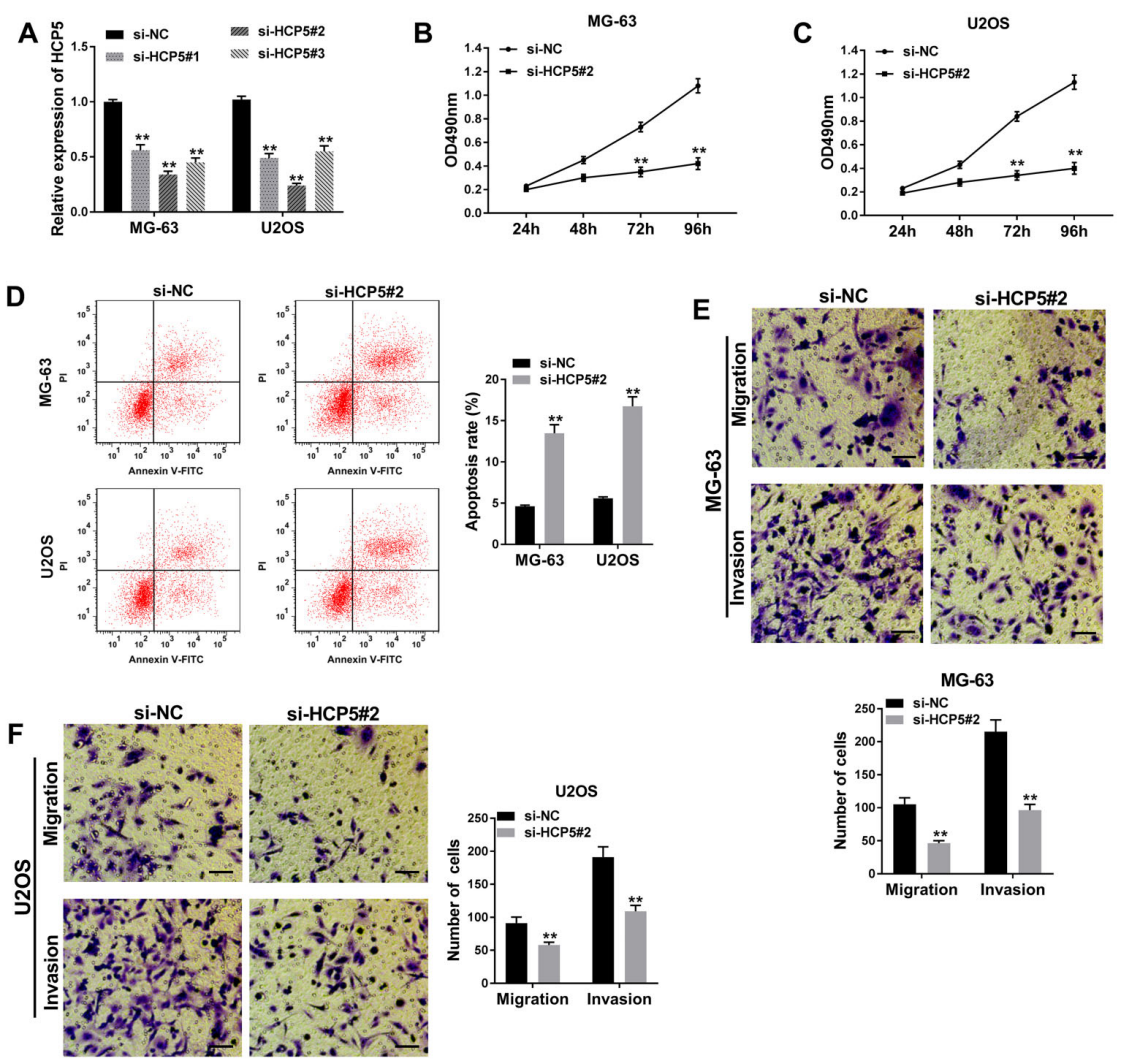
HCP5 knockdown suppressed proliferation, migration, and invasion, and promoted apoptosis in osteosarcoma (OS) cells. **A**, The expression of HCP5 in MG-63 and U2OS cells transfected with si-NC, si-HCP5#1, si-HCP5#2, or si-HCP5#3 was measured by qRT-PCR. **B** and **C**, Cell proliferative capacity was evaluated by MTT assay. **D**, Cell apoptosis was detected 48 h after transfection by flow cytometry. **E** and **F**, Cell migration and invasion were confirmed by transwell assay in MG-63 and U2OS cells (100×, scale bar: 50 μm). Data are reported as means±SD. **P<0.01 (Student's *t*-test and ANOVA).

At the beginning, the efficiency of HCP5 knockdown was detected by qRT-PCR. As shown in Figure 2A, the expression of HCP5 in MG-63 and U2OS of 3 groups (si-HCP#1, si-HCP#2, and si-HCP#3) was down-regulated, and the highest decline in expression was in si-HCP#2, suggesting that the knockdown efficiency of si-HCP#2 was the highest. Hence, si-HCP#2 was chosen for the following assays. MTT assay showed that cell proliferation was inhibited in MG-63 and U2OS transfected with si-HCP5#2 compared with si-NC (Figure 2B and C). Flow cytometry indicated that si-HCP5#2 transfection was notably beneficial to cell apoptosis rate in OS cell lines (Figure 2D). Unsurprisingly, transwell assay identified that cell migration and invasion were restrained when HCP5 was down-regulated (Figure 2E and F). All data suggested that knockdown of HCP5 had a tumor-suppressive role in OS.

### HCP5 regulated the expression of miR-101 by targeting miR-101

**Figure 3:**
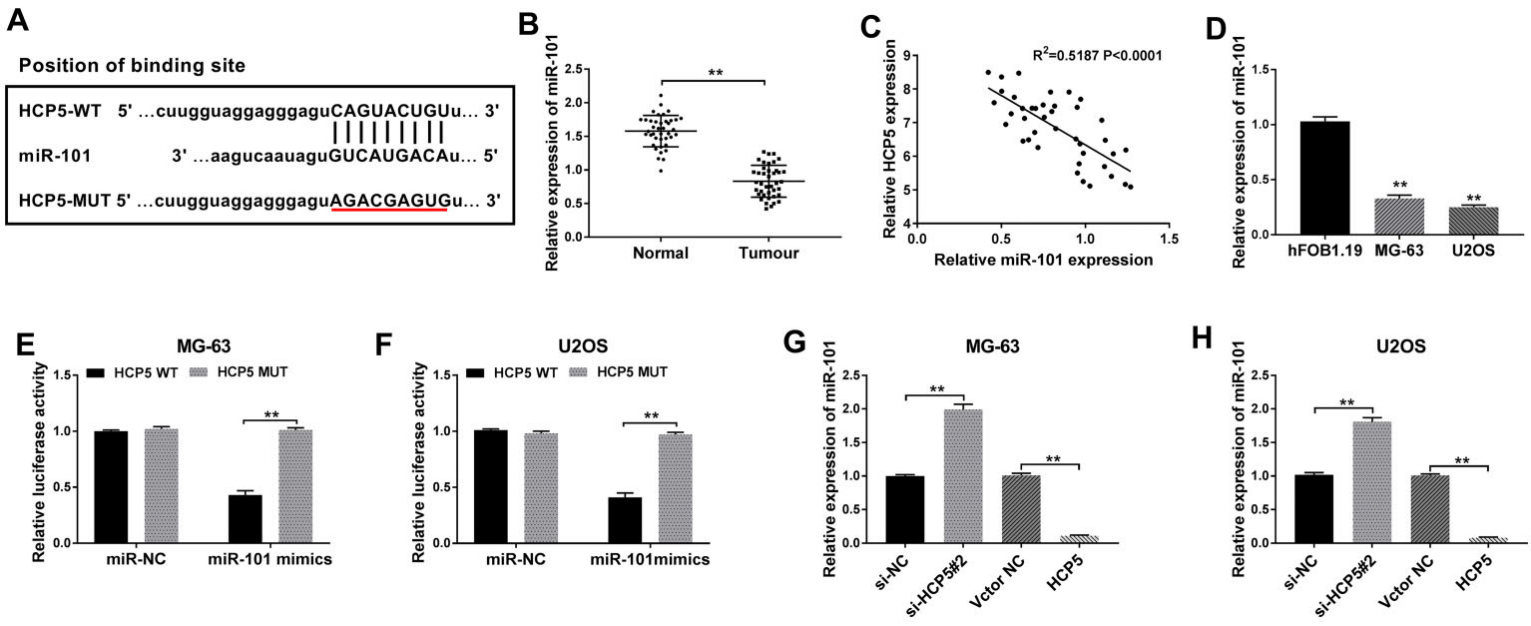
HCP5 directly targeted miR-101. **A**, The online software miRcode predicted the putative binding sites between HCP5 and miR-101. **B**, The expression of miR-101 was exhibited in osteosarcoma (OS) tissues and adjacent normal tissues. **C**, Correlation between the expression of HCP5 and miR-101 in OS tissues. **D**, The expression of miR-101 was measured in OS cell lines MG-63 and U2OS and normal cells hFOB 1.19. **E** and **F**, Luciferase reporter assay was performed to confirm the interaction between HCP5 and miR-101 in MG-63 and U2OS cells. **G** and **H**, Relative expression of miR-101 was examined in MG-63 and U2OS cells transfected with si-NC (negative control), si-HCP5#2, vector NC, or HCP5. Data are reported as means±SD. **P<0.01 (Student's *t*-test and ANOVA).

To investigate the interaction between HCP5 and miR-101, we first predicted the potential binding sites of HCP5 and miR-101 through miRcode software (http://www.mircode.org) (Figure 3A). Then, this prediction was confirmed by verification of miR-101 expression. Results from qRT-PCR indicated that the expression of miR-101 in tumor tissues was lower than that in normal tissues (Figure 3B). We also saw that the expression of miR-101 was negatively correlated with HCP5 expression in OS tissues (Figure 3C). Similarly, miR-101 expression declined in MG-63 and U2OS cells compared with that in hFOB 1.19 cells (Figure 3D). To further confirm the relationship, we performed luciferase reporter assay, and the data showed that miR-101 mimics significantly reduced luciferase activity in MG-63 and U2OS cells with HCP5-WT transfection, while cotransfection of miR-101 mimics and HCP5-MUT did not change the luciferase activity compared to miR-NC (Figure 3E and F). In addition, expression of miR-101 was significantly enhanced in MG-63 and U2OS cells transfected with si-HCP5#2 compared to si-NC, while miR-101 expression was significantly weakened in cells transfected with HCP5 compared to vector NC (Figure 3G and H). These data indicated that HCP5 regulated the expression of miR-101 through targeting miR-101 directly.

### MiR-101 deficiency reversed the effects of HCP5 knockdown on cell proliferation, apoptosis, migration, and invasion

**Figure 4:**
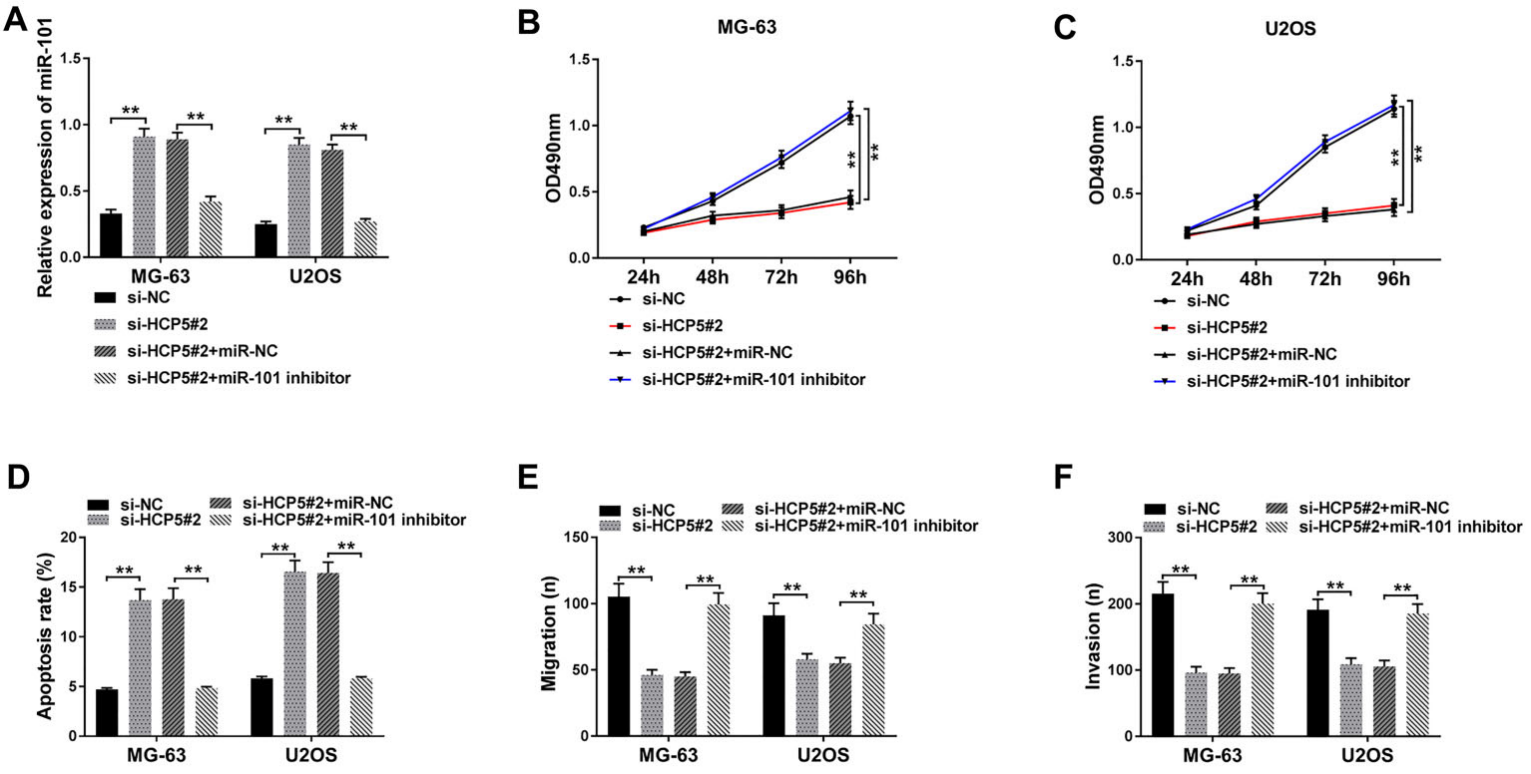
Depletion of miR-101 could reverse the impact on cell proliferation, apoptosis, migration, and invasion caused by HCP5 knockdown in osteosarcoma (OS) cells. **A**, The expression of miR-101 in MG-63 and U2OS cells transfected with si-NC (negative control), si-HCP5#2, si-HCP5#2+miR-NC, or si-HCP5#2+miR-101 inhibitor was measured by qRT-PCR. **B** and **C**, Cell proliferation was assessed at 24, 48, 72, and 96 h after transfection by MTT assay in MG-63 and U2OS cells. **D**, Cell apoptosis was analyzed by flow cytometry. **E** and **F**, Transwell assay was used for cell migration and invasion analysis in MG-63 and U2OS cells. Data are reported as means±SD. **P<0.01 (Student's *t*-test and ANOVA).

To further determine the biological interaction between HCP5 and miR-101 in OS cells, si-HCP5#2, si-NC, si-HCP5#2+miR-101 inhibitor, and si-HCP5#2+miR-NC were transfected into MG-63 and U2OS cells. First, we detected the expression of miR-101 and found that it was up-regulated in MG-63 and U2OS transfected with si-HCP5#2, while miR-101 expression was significantly decreased in cells transfected with si-HCP5#2+miR-101 inhibitor (Figure 4A). Then, MTT assay revealed that the decreased cell viability of MG-63 and U2OS cells caused by HCP5 knockdown was reversed by miR-101 inhibition (Figure 4B and C). The increase of HCP5 knockdown on cell apoptosis was abolished by miR-101 inhibition (Figure 4D). Transwell assay showed that cotransfection of si-HCP5#2+miR-101 inhibitor reversed suppressive effects on cell migration and invasion caused by HCP5 knockdown alone (Figure 4E and F). These data suggested that HCP5 accelerated the development of OS through regulating miR-101 expression.

### EPHA7 was a specific target of miR-101

**Figure 5:**
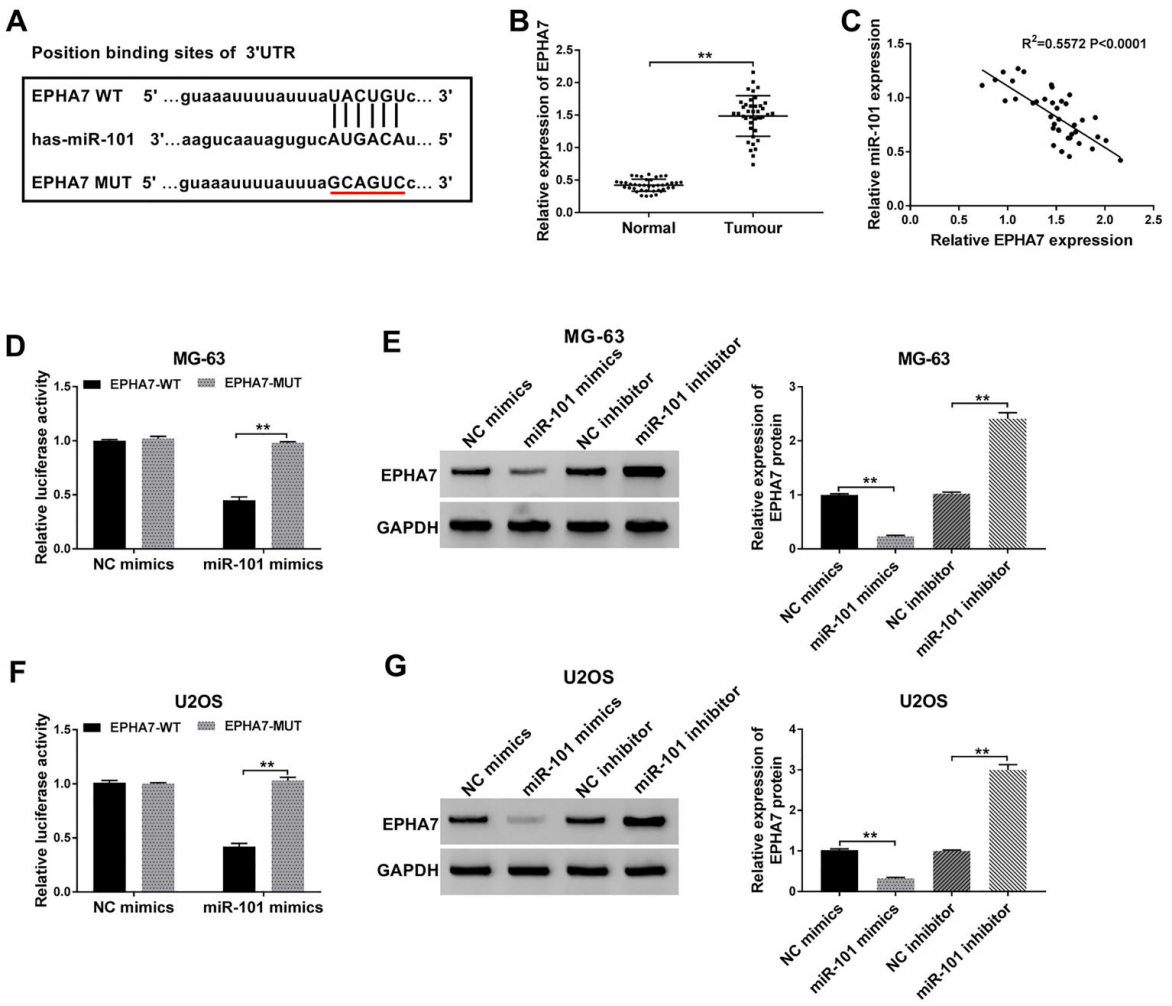
EPHA7 was a target of miR-101. **A**, Predicted binding sites between miR-101 and EPHA7 3'UTR. **B**, The expression of EPHA7 was measured through qRT-PCR assay in osteosarcoma (OS) tissues and adjacent normal tissues. **C**, Correlation analysis between miR-101 expression and EPHA7 expression in OS tissues. **D** and **F**, Relative luciferase activity was measured in MG-63 and U2OS cells cotransfected with miR-101 mimics and EPHA7-WT (wild-type) or EPHA7-MUT (mutant). **E** and **G**, EPHA7 protein expression level was observed by western blot in MG-63 and U2OS cells with NC (negative control) mimics, miR-101 mimics, NC inhibitor, or miR-101 inhibitor transfection. Data are reported as means±SD. **P<0.01 (Student's *t*-test and ANOVA).

To determine the relationship between miR-101 and EPHA7, the possible binding sites between miR-101 and EPHA7 3′UTR were predicated using TargetScan software. EPHA7 was found to be a putative target of miR-101 (Figure 5A). Data from qRT-PCR showed that the expression of EPHA7 was abundant in OS tissues compared with normal tissues (Figure 5B). A negative correlation was found between miR-101 expression and EPHA7 expression in OS tissues (Figure 5C). Luciferase reporter assay indicated that the luciferase activity of MG-63 and U2OS cells transfected with EPHA7-WT and miR-101 mimics was significantly depleted compared with miR-NC, while the luciferase activity of cells transfected with EPHA7-MUT and miR-101 mimics was not changed compared to miR-NC (Figure 5D and F). Additionally, the protein level of EPHA7 was significantly increased in MG-63 and U2OS cells transfected with miR-101 inhibitor but reduced rapidly in cells transfected with miR-101 mimics (Figure 5E and G). These data showed that miR-101 directly targeted the 3′UTR of EPHA7 to mediate EPHA7 expression.

### EPHA7 downregulation reversed the effects of miR-101 inhibition in OS cells

**Figure 6:**
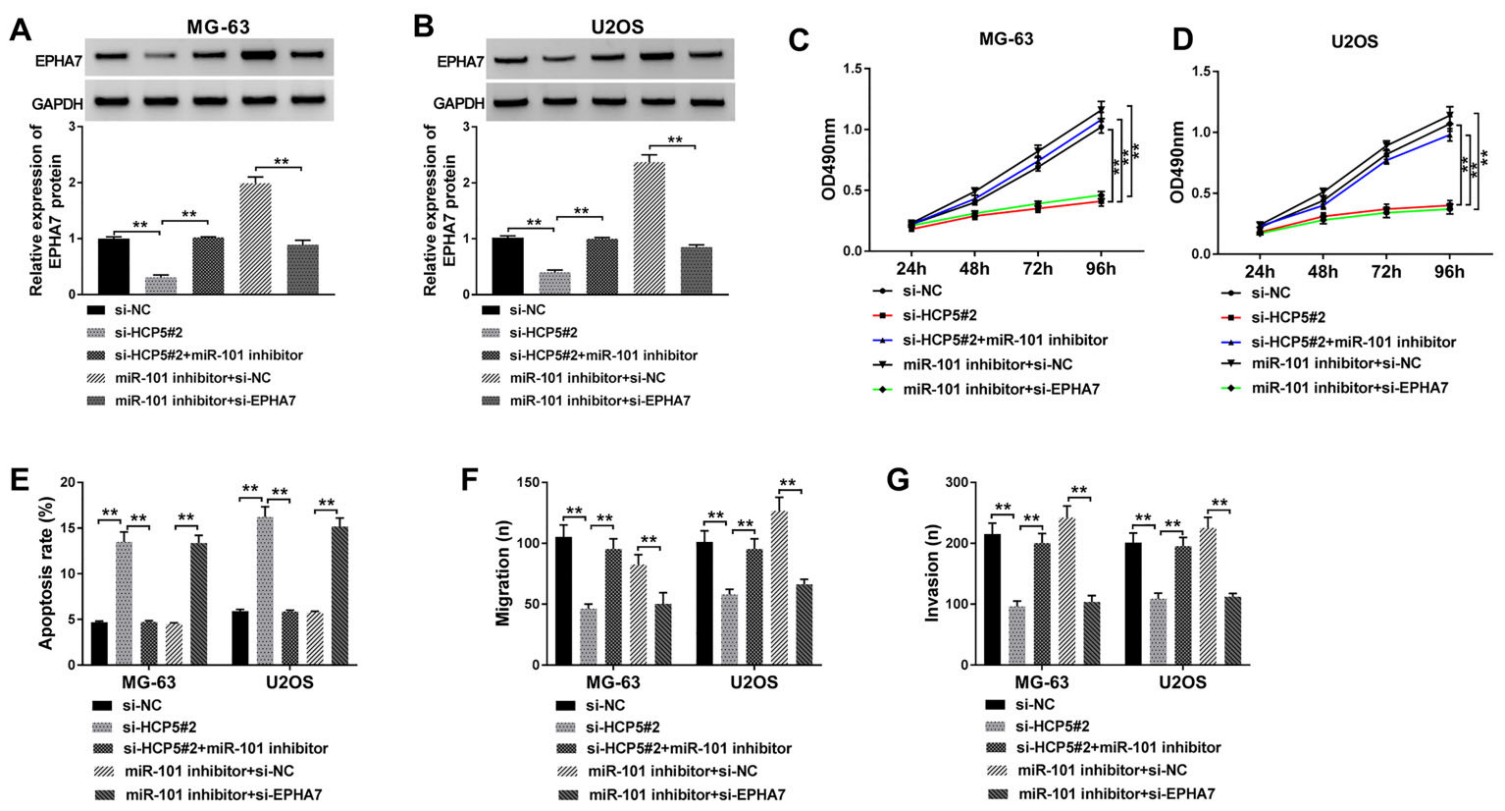
HCP5 regulated cell proliferation, apoptosis, migration, and invasion in osteosarcoma (OS) cells by controlling the expression of EPHA7 via targeting miR-101. **A** and **B**, Relative protein expression level of EPHA7 was determined by western blot in MG-63 and U2OS cells transfected with si-NC (negative control), si-HCP5#2, si-HCP5#2+miR-101 inhibitor, miR-101 inhibitor+si-NC, and miR-101 inhibitor+si-EPHA7. **C** and **D**, Cell proliferation was measured by MTT assay in MG-63 and U2OS cells containing diverse transfection. **E**, Cell apoptosis was examined using flow cytometry. **F** and **G**, Cell migration and invasion were detected in MG-63 and U2OS cells after transfection by transwell assay. Data are reported as means±SD. **P<0.01 (ANOVA).

To further define the molecular mechanism of HCP5 in OS cells, si-HCP5#2, si-NC, si-HCP5#2+miR-101 inhibitor, miR-101 inhibitor+si-EPHA7, and miR-101 inhibitor+si-NC were transfected into MG-63 and U2OS cells. Western blot analysis showed that EPHA7 expression was significantly decreased in cells transfected with si-HCP5#2 compared with si-NC, whereas EPHA7 expression was restored in cells transfected with si-HCP5#2+miR-101 inhibitor. In addition, the expression of EPHA7 in cells transfected with miR-101 inhibitor+si-EPHA7 was clearly lower than that transfected with miR-101 inhibitor+si-NC (Figure 6A and B), suggesting HCP5 directly regulated the expression of EPHA7 by targeting miR-101. Functionally, MTT assay characterized that miR-101 inhibition could reverse the effects of HCP5 knockdown to promote proliferation, and EPHA7 downregulation could reverse the effects of miR-101 inhibition to impair proliferation (Figure 6C and D). On the contrary, miR-101 inhibition could reverse the effects of HCP5 knockdown to decrease the apoptotic rate, and EPHA7 downregulation could reverse the effects of miR-101 inhibition and increase the apoptotic rate (Figure 6E). Moreover, transwell assay showed that miR-101 deficiency partly abolished the effects of HCP5 knockdown to promote migration and invasion, and EPHA7 downregulation partly abolished the effects of miR-101 deficiency to block migration and invasion (Figure 6F and G). The above expression analysis and reverse experiment indicated that HCP5 contributed to OS malignant development by regulating EPHA7 expression via sponging miR-101.

## Discussion

In the past 25 years, new chemotherapy regimens for OS have failed to improve long-term survival rates above 65∼70% (1). Tumors have a high tendency to metastasize, one of the most common causes of death associated with cancer (21). Currently, multiple studies have reported that lncRNAs play specific roles in the development and metastasis of cancer, so characterization of cancer-related lncRNAs and their target miRNAs involved in carcinogenesis attract more attention.

In our study, we demonstrated that HCP5 was up-regulated in OS tissues and cell lines. Down-regulation of HCP5 suppressed OS cell proliferation, invasion, and migration. HCP5 is a well-known lncRNA that is associated with numerous cancers, such as human triple-negative breast cancer (22), glioma (7), and hepatitis C virus-associated hepatocellular carcinoma (23). Several studies have demonstrated that HCP5 expression is enhanced in tumor tissues, including lung adenocarcinoma (24), anaplastic thyroid cancer (25), and colorectal cancer (26). Interestingly, the result in this paper was consistent with previous studies, suggesting that HCP5 universally had an oncogenic role in cancers. However, the molecular mechanisms by which HCP5 functions in cancer are complex and diverse, thus our research focused on the possible mechanisms of HCP5 in OS.

We found that HCP5 could target miR-101 directly. The function of miR-101 in OS has been partly reported. Jiang et al. (27) showed that miR-101 was significantly down-regulated in common OS cell lines, including MG63, U2OS, and OS732. Additionally, up-regulation of miR-101 inhibited cell viability, migration, and invasion but promoted apoptosis. Lin et al. (28) proved that miR-101 is down-regulated in OS and could suppress OS cell proliferation and invasion by directly targeting zinc finger E-box binding homeobox 2 (ZEB2). Zhang et al. (29) observed that miR-101 functions as a tumor suppressor in OS because it depletes cell migration and invasion by targeting enhancer of zeste homolog 2 (EZH2). In this study, we demonstrated that miR-101 had a lower expression in OS tissues and cells, and the effects of HCP5 down-regulation on cell proliferation, apoptosis, migration, and invasion could be reversed by miR-101 inhibitor. This was in accordance with the above-mentioned research. The dysregulated expression of miRNAs is involved in many types of cancers, acting at the post-transcriptional level by inhibiting the translation of protein-coding genes or inducing mRNA dysregulation, regulating various biological processes.

EPHA7 is a target of miR-101, which was confirmed in this report. We further observed that EPHA7 expression was reinforced in OS tissues, and low expression of miR-101 accelerated OS cell proliferation, migration, and invasion through targeting EPHA7. EPHA7 has been reported in several cancers, such as non-small cell lung cancer (18), human laryngeal cancer (19), and adenocarcinoma (30). Interestingly, the expression of EPHA7 in these cancers was intensified, and our results were similar to these studies. However, other studies report the opposite showing that EPHA7 may act as a tumor suppressor. For example, EPHA7 inhibits malignant growth of prostate cancer by targeting the PI3K/Akt signaling pathway (31). Another report supported that EPHA7 might be a new tumor suppressor gene for 6q deletions in T-cell lymphoblastic leukemia/lymphoma (32). The molecular mechanism of EPHA7 in different cancers is variable, and EPHA7 may exert tissue-specific or cell-specific functions. However, our observation was concordant with the study showing that EPHA7 expression was up-regulated in OS tissues (33), suggesting EPHA7 exerted a carcinogenic role in OS.

Taken together, our findings demonstrated that HCP5 expression was enhanced in OS tissues and cells, and HCP5 aggravated OS cell proliferation, migration, and invasion by promoting the expression of EPHA7 via targeting miR-101 competitively. Our study provided new insights into the role of HCP5 in OS and a novel mechanism of the HCP5/miR-101/EPHA7 axis in OS development. HCP5 may be a potential prognostic biomarker and gene therapeutic agent for OS treatment.

## References

[1] Lindsey BA, Markel JE, Kleinerman ES (2017). Osteosarcoma overview. Rheumatol Ther.

[2] Luetke A, Meyers PA, Lewis I, Juergens H (2014). Osteosarcoma treatment-where do we stand? A state of the art review. Cancer Treat Rev.

[3] Deng G, Sui G (2013). Noncoding RNA in oncogenesis: a new era of identifying key players. Int J Mol Sci.

[4] Huang T, Alvarez A, Hu B, Cheng SY (2013). Noncoding RNAs in cancer and cancer stem cells. Chin J Cancer.

[5] Ponting CP, Oliver PL, Reik W (2009). Evolution and functions of long noncoding RNAs. Cell.

[6] Liang L, Xu J, Wang M, Xu G, Zhang N, Wang G (2018). LncRNA HCP5 promotes follicular thyroid carcinoma progression via miRNAs sponge. Cell Death Dis.

[7] Teng H, Wang P, Xue Y, Liu X, Ma J, Cai H (2016). Role of HCP5-miR-139-RUNX1 feedback loop in regulating malignant behavior of glioma cells. Mol Ther.

[8] Yu Y, Shen H, Fang D, Meng Q, Xin Y (2018). LncRNA HCP5 promotes the development of cervical cancer by regulating MACC1 via suppression of microRNA-15a. Eur Rev Med Pharmacol Sci.

[9] Bartel DP (2004). MicroRNAs: genomics, biogenesis, mechanism, and function. Cell.

[10] Rupaimoole R, Calin GA, Lopezberestein G, Sood AK (2016). miRNA deregulation in cancer cells and the tumor microenvironment. Cancer Discov.

[B11] Ha M, Kim VN (2014). Regulation of microRNA biogenesis. Nat Rev Mol Cell Biol.

[B12] Lin S, Gregory RI (2015). MicroRNA biogenesis pathways in cancer. Nat Rev Cancer.

[13] Rupaimoole R, Slack FJ (2017). MicroRNA therapeutics: towards a new era for the management of cancer and other diseases. Nat Rev Drug Discov.

[14] Huang Y, Zou Y, Lin L, Ma X, Zheng R (2019). miR-101 regulates cell proliferation and apoptosis by targeting KDM1A in diffuse large B cell lymphoma. Cancer Manag Res.

[15] Chu P, Wang Q, Wang Z, Gao C (2019). Long non-coding RNA highly up-regulated in liver cancer protects tumor necrosis factor-alpha-induced inflammatory injury by down-regulation of microRNA-101 in ATDC5 cells. Int Immunopharmacol.

[16] Gonzales-Aloy E, Connerty P, Salik B, Liu B, Woo AJ, Haber M (2019). miR-101 suppresses the development of MLL-rearranged acute myeloid leukemia. Haematologica.

[17] Janes PW, Bettina G, Lakmali A, Eva N, Hii LL, Anneloes M (2011). Eph receptor function is modulated by heterooligomerization of A and B type Eph receptors. J Cell Biol.

[18] Liu M, Zhou K, Cao Y (2016). MicroRNA-944 affects cell growth by targeting EPHA7 in non-small cell lung cancer. Int J Mol Sci.

[19] Xiang C, Lv Y, Wei Y, Wei J, Miao S, Mao X (2015). Effect of EphA7 silencing on proliferation, invasion and apoptosis in human laryngeal cancer cell lines Hep-2 and AMC-HN-8. Cell Physiol Biochem.

[20] Li R, Sun Y, Jiang A, Wu Y, Li C, Jin M (2016). Knockdown of ephrin receptor A7 suppresses the proliferation and metastasis of A549 human lung cancer cells. Mol Med Rep.

[21] Botter SM, Neri D, Fuchs B (2014). Recent advances in osteosarcoma. Curr Opin Pharmacol.

[22] Wu J, Chen H, Ye M, Wang B, Zhang Y, Sheng J (2019). Downregulation of long noncoding RNA HCP5 contributes to cisplatin resistance in human triple-negative breast cancer via regulation of PTEN expression. Biomed Pharmacother.

[23] Lange CM, Bibert S, Dufour JF, Cellerai C, Cerny A, Heim MH (2013). Comparative genetic analyses point to HCP5 as susceptibility locus for HCV-associated hepatocellular carcinoma. J Hepatol.

[24] Jiang L, Wang R, Fang L, Ge X, Chen L, Zhou M (2019). HCP5 is a SMAD3-responsive long non-coding RNA that promotes lung adenocarcinoma metastasis via miR-203/SNAI axis. Theranostics.

[25] Chen J, Zhao D, Meng Q (2019). Knockdown of HCP5 exerts tumor-suppressive functions by up-regulating tumor suppressor miR-128-3p in anaplastic thyroid cancer. Biomed Pharmacother.

[26] Yang C, Sun J, Liu W, Yang Y, Chu Z, Yang T (2019). Long noncoding RNA HCP5 contributes to epithelial-mesenchymal transition in colorectal cancer through ZEB1 activation and interacting with miR-139-5p. Am J Transl Res.

[27] Jiang R, Zhang C, Liu G, Gu R, Wu H (2017). MicroRNA-101 inhibits proliferation, migration and invasion in osteosarcoma cells by targeting ROCK1. Am J Cancer Res.

[28] Lin H, Zheng X, Lu T, Gu Y, Zheng C, Yan H (2019). The proliferation and invasion of osteosarcoma are inhibited by miR-101 via targeting ZEB2. Biosci Rep.

[29] Zhang K, Zhang Y, Ren K, Zhao G, Yan K, Ma B (2014). MicroRNA-101 inhibits the metastasis of osteosarcoma cells by downregulation of EZH2 expression. Oncol Rep.

[30] Liu DC, Yang ZL (2013). MTDH and EphA7 are markers for metastasis and poor prognosis of gallbladder adenocarcinoma. Diagn Cytopathol.

[31] Li S, Wu Z, Ma P, Xu Y, Chen Y, Wang H (2017). Ligand-dependent EphA7 signaling inhibits prostate tumor growth and progression. Cell Death Dis.

[32] López-Nieva P, Vaquero C, Fernández-Navarro P, González-Sánchez L, Villa-Morales M, Santos J (2012). EPHA7, a new target gene for 6q deletion in T-cell lymphoblastic lymphomas. Carcinogenesis.

[33] Wu X, Yan L, Liu Y, Xian W, Wang L, Ding X (2017). MicroRNA-448 suppresses osteosarcoma cell proliferation and invasion through targeting EPHA7. PloS One.

